# Walking on Tiptoes: Digital Pads Deserve Increased Attention When Scoring Footpad Dermatitis as an Animal Welfare Indicator in Turkeys

**DOI:** 10.3389/fvets.2020.613516

**Published:** 2021-01-06

**Authors:** Jenny Stracke, Nina Volkmann, Franziska May, Stefanie Döhring, Nicole Kemper, Birgit Spindler

**Affiliations:** ^1^Institute for Animal Hygiene, Animal Welfare and Animal Behavior, University of Veterinary Medicine Hannover, Foundation, Hannover, Germany; ^2^Faculty of Agricultural Sciences and Landscape Architecture, Osnabrück University of Applied Sciences, Osnabrück, Germany

**Keywords:** footpad dermatitis, FPD, turkey, digital pad, animal welfare, welfare indicator

## Abstract

Animal welfare is one of the most challenging issues in modern farm animal husbandry. Animal welfare indicators can be used to monitor welfare on farms or at slaughterhouses, with footpad dermatitis (FPD) being one of the most important indicators used in turkeys. Up to now, the severity of FPD has been measured by evaluating the size of altered lesions on the metatarsal pad of birds. However, such lesions are not only found on the metatarsal pads, but alterations can also occur on the digital pads of the animals, the latter is not included in the European standard scoring systems for turkeys so far. The aim of the present study was to give a detailed outline of alterations on the digital pads of turkeys and associate their occurrence to a standardly used five-point scoring system, which is based on alterations of the metatarsal pad only. Therefore, pictures of 500 feet of turkeys from 16 flocks at the end of the fattening phase were taken, using an automatic camera system. Based on these pictures, alterations on the digits were scored according to different parameters (lesions, swellings, and number of affected digits). Furthermore, detailed measurements were conducted using an imaging software. Results were compared with a standardly used five-point scoring system (standard FPD scoring system), based on the metatarsal pad as reference. Results provide no equivalence in occurrence and severity of alterations on the metatarsal pads compared to those found on the digits. Pathologic alterations on the digits were already present at standard FPD scoring level 0; no differentiation became obvious between the higher scoring levels 2–4. Strong correlations were found when comparing percentage of alterations of the standard FPD scoring system to those of a system including alterations on the digits and the metatarsal pad, using the total foot as a reference (r_p_ = 0.9, *p* < 0.001). This was the first study conducting a detailed analysis of alterations on the digits of turkeys. In conclusion, results of this study show that including the evaluation of alterations on digits could refine the present FPD scoring system, especially when using FPD as an animal welfare indicator.

## Introduction

Animal welfare is not only of increasing concern in public but also gains importance in scientific and animal husbandry fields. In turkeys, FPD is one of the most frequently used welfare indicators providing information on animal health and well-being. This is due to the fact that FPD is closely associated with the husbandry system, with litter quality being the major determinant for this pathology (1–4). Furthermore, the manifestation of FPD is easy and quick to assess, implying its advantage to be utilized in various welfare assessment schemes, which are applied on-farm to measure animal welfare (5, 6).

FPD is described as a contact dermatitis of the plantar surface of birds' feet (7), which can show a wide range of characteristics. It occurs with different severity grades and can affect the surface but also subjacent structures (7, 8). Evaluation of severity is generally performed using scoring systems, categorizing the different incidents according to a subjective assessment of the size of the alteration. There are different scoring systems, which can be used in turkeys (1, 9, 10). However, a major breakthrough came in 2008, with Hocking et al. (11) proposing a standard scoring system to be used Europe-wide. This is especially important, as the monitoring of FPD at the slaughterhouse and on-farm is nowadays an accepted tool all over Europe and in the United States [see (9) for the United Kingdom and the United States; (11, 12) for Europe]. In order to ensure reliability (which is essential in a scientific context, but also plays a great role when focusing on international market competition), such a system, especially when practically used at the slaughterhouse, should be clearly defined, results should be repeatable between different classifiers and it should be quick and easy to use. The five-point scoring system proposed by Hocking et al. (11) fulfills all these criteria (11, 13, 14).

Nonetheless, this system also has its limitations, which become particularly important when using FPD not only as a benchmark system, but also as a welfare indicator, which is the current trend. In regard to animal welfare and to animal welfare legislation [(15), Article 7], unnecessary suffering should be avoided. Ulcerations can be considered as highly relevant in this context, as they are most likely to induce pain (1, 16, 17). Ulcerations are described as a loss of the epidermis, usually associated with an inflammatory reaction (18). According to a study by Stracke et al. (14), there is a link between the occurrence of ulcerations and the size of the lesion on the metatarsal pad, findings which are in agreement with Toppel et al. (13). Similar results were observed for broiler chickens (19, 20). However, using the standard scoring system of Hocking et al. (11), the study by Stracke et al. (14) also showed that no differences in severity of ulcerations were found between the higher scoring levels (scoring levels 2, 3, and 4). Furthermore, other incidents, such as re-epithelialized granulation tissue and chronic inflammation processes, were not to be found to be linked to the size of the lesion either. Stracke et al. (14) therefore raised the question as to whether the standardly used scoring system should be revised, at least with regard to the implementation of FPD as an animal welfare indicator. The present study takes a similar approach, albeit concentrating on another aspect, here in particular questioning the reference values used for evaluating FPD. Using the standard FPD scoring system, the metatarsal pad and its alterations are considered to represent incidence thereof. However, birds affected by FPD not only show alterations on the metatarsal pads. Alterations can also be found on the digital pads of the animals, as mentioned in various studies (1, 3, 7). Some existing studies state that the occurrence of FPD at the digits is most likely accompanied by higher scoring levels of measurements related to the metatarsal pad (21, 22). Up to now, there is no systematic study describing the development of lesions on the digital pads of turkeys. Consequently, the above-mentioned assumptions are not scientifically verified yet. Hocking et al. (11) also state that there are similar alterations at the toes compared to the ones found on the metatarsal pads and therefore, in order to obtain simplicity, they refrain from including the digits in their scoring systems. With regard to visual scoring systems, this might well be the case. However, with new forms of automatic technology for evaluating FPD, this approach might be worth reconsidering. Precision Livestock Farming (PLF) is rapidly developing in the poultry sector worldwide (23), thus offering opportunities to increase the efficiency and sustainability of farming and production and to improve animal health and welfare (24). Image analysis seems to be a promising approach to automatically evaluate FPD at the slaughterhouse, first systems of which have already been used for broilers (25, 26). In German slaughterhouses, a similar technique is employed for turkeys (13), with the classification of severity levels being based on the standard scoring system of Hocking et al. (11). In this latter study, the authors state that integrating alterations on the digits might improve the quality of FPD evaluation.

Therefore, in order to improve the standard FPD scoring system for usage in animal welfare measurements, the present study aimed to provide a detailed description of the lesions on digits. More specifically, the aim was to evaluate a potential linkage between lesions on the metatarsal and digital pads in turkey feet. According to current literature, the hypothesis is, that, with rising severity of FPD on the metatarsal pad, an increase in severity of alterations on digitals can be found.

## Methods

**Figure 1:**
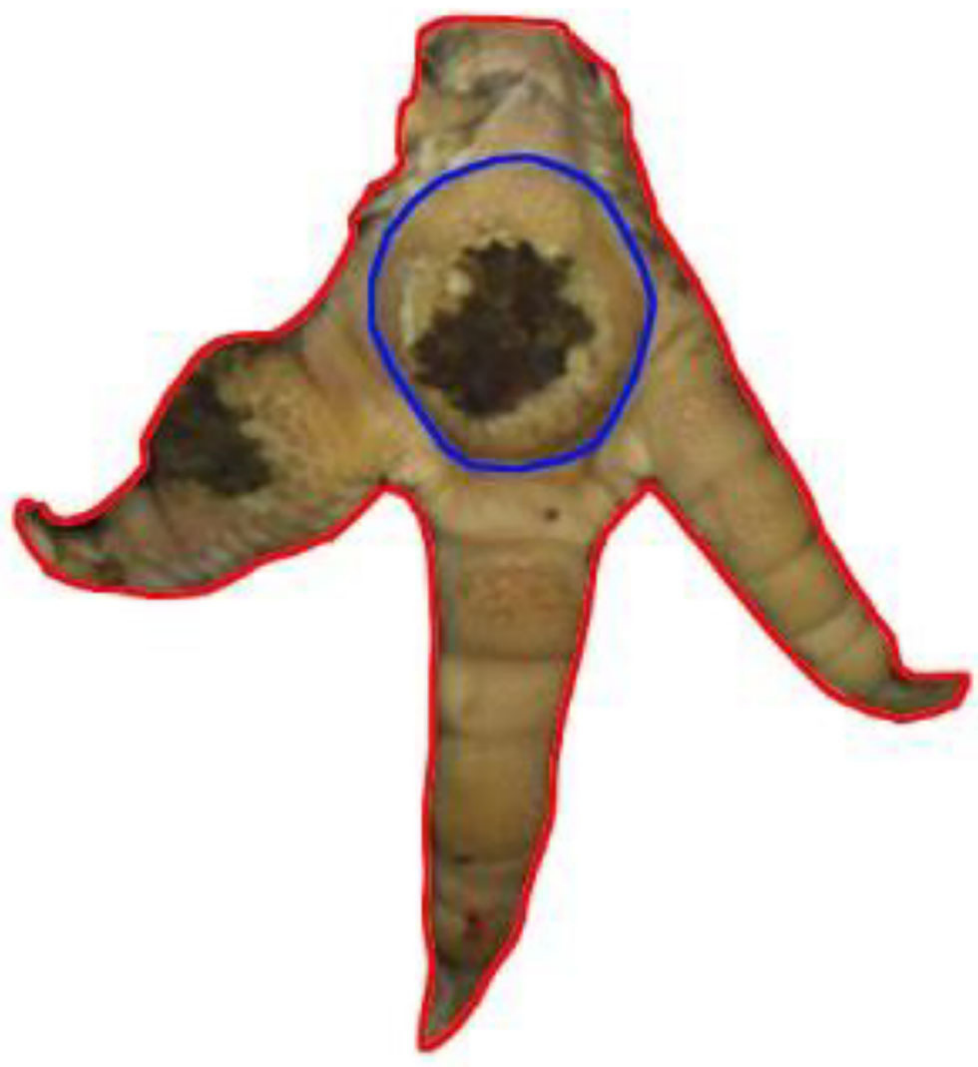
References for scoring. Total foot = RA1 is marked in red; Metatarsal pad = RA2 is marked in blue.

Turkey feet (B.U.T. 6, Aviagen Turkeys Ltd., Tattenhall, UK) were monitored at a German slaughterhouse at the end of the fattening phase. The outlined study was part of a larger project with the focus to validate the accuracy of automatic systems for the evaluation of FPD in turkeys at the slaughterhouse. The sample size used for the present study resulted from the original research question. Therefore, no a priori power analysis was conducted. In total, pictures of 16 flocks of male animals were taken using an automatic camera system (CLK GmbH; Turkey Check V1.0, Altenberge, Germany), which was a fixed part of the slaughter line used for continuously monitoring FPD. The camera system was installed at the end of the slaughter line, where feet are already separated from the body. It takes pictures of each foot passing the camera; one foot per pair of feet is used for further processing. Typically, the left foot per pair is used. However, if the left foot cannot be detected clearly, the camera switches to the right foot, respectively. The system, which is equipped with a software, is based on 2-D-RGB-image analysis and processing. Feet were detected using the contrast to a dark background. The software then checks the metatarsal pad for discoloration (darker areas). The metatarsal pad was defined as the difference between the foot and the digits, using the contrast between the brighter colors of the skin at the digits vs. the dark color of the background in the interspaces between the digits, leaving out the digits, putting a circle around the rest (see Figure 1).

From the above mentioned flocks, 500 feet were picked in a pseudorandomized order, including 100 feet per scoring level of a five-point classification system [standard FPD scoring system, (11); see Table 1]. Feet were picked according to the scoring of the above mentioned camera system. Therefore, only the evaluated foot per pair was included in the sample, which could be either the right or the left one.

**Table 1:** Scoring system for the footpad dermatitis, based on alterations on the metatarsal pad adapted from Hocking et al. (13) (standard FPD scoring system) (pictures taken by Jenny Stracke).

**Score 0**	**Score 1**	**Score 2**	**Score 3**	**Score 4**
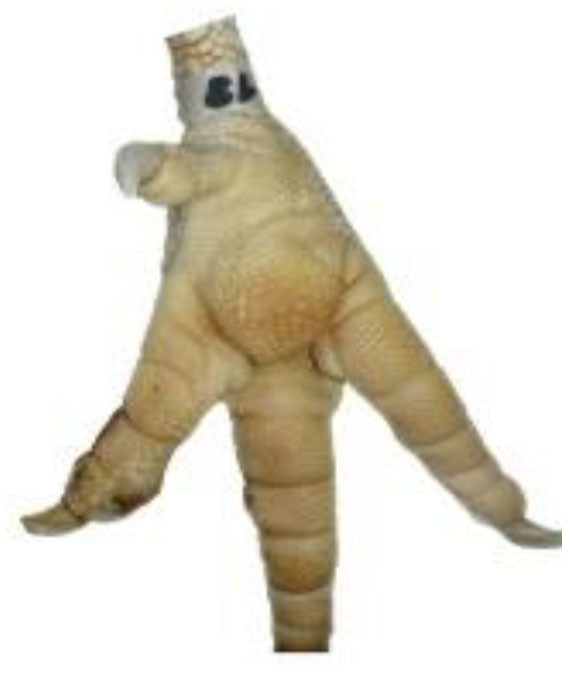	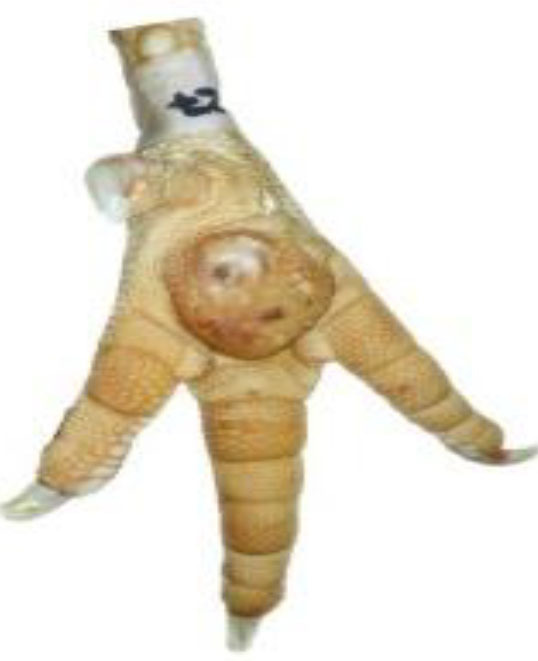	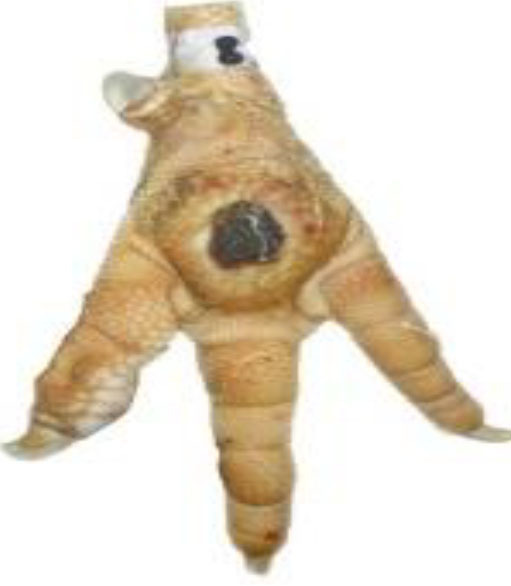	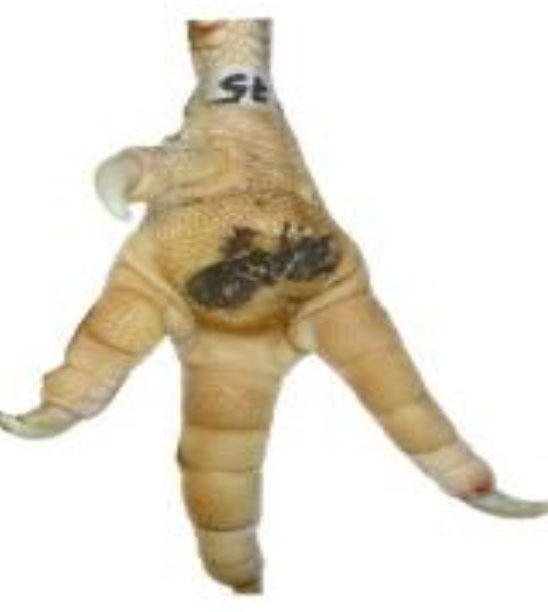	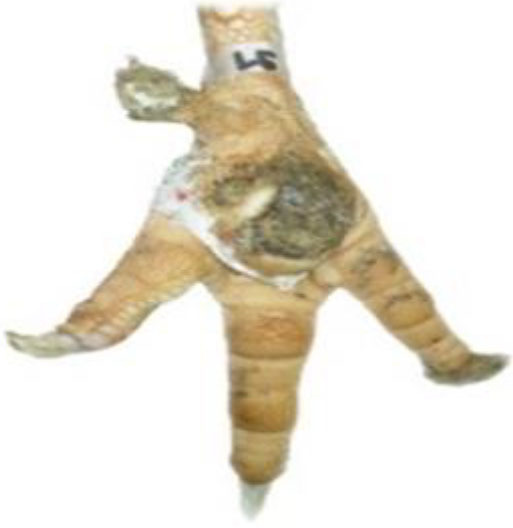
Intact foot	Small, punctual alterations, <10% of the footpad	Altered lesion covers ≤25% of the footpad	Altered lesion covers ≤50% of the footpad	Altered lesion covers more than 50% of the footpad

All pictures used for the analysis were verified manually regarding the performance of the automatic system. In a first step, a manual observer checked if the metatarsal pad and the alteration on the footpad had been correctly identified; in a second step, all footpads were scored manually, scoring levels (manual vs. automatic) having to be identical. Feet used for further analysis had to fulfill all of these selection criteria.

### Scoring System FPD Metatarsal Pads

The definition of the different scoring levels can be found in Table 1. This scoring system is hereafter referred to as standard FPD scoring system.

### Scoring System FPD Digital Pads

Only digital pads 2–4 were evaluated. This was due to practical reasons, as the suspension of feet in the slaughter line did not allow for the detection of the first digit. Each digital pad (DP) was scored separately, using a five-scale score to evaluate the altered lesion (Table 2) and a three-scale score was used, providing information on the severity grade of swelling (Table 3). Altered lesions were scored in relation to the different segments per digit. Furthermore, the number of affected digits was counted. The severity grade of swelling was evaluated using the corresponding digit of the respective (unaffected) second foot of each pair of feet as a reference.

**Table 2:** Scoring system for the digital pads: Altered lesion on the digital pad.

**Score 0**	**Score 1**	**Score 2**	**Score 3**	**Score 4**
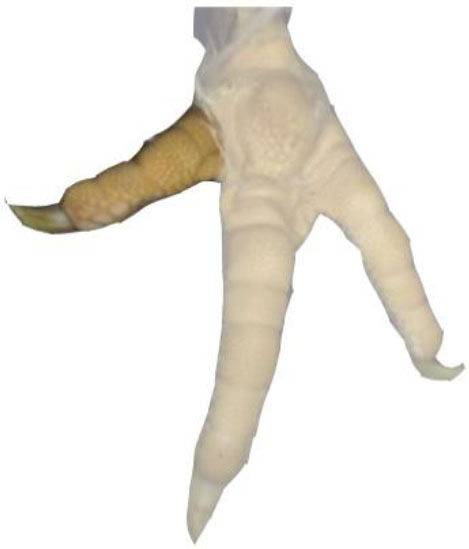	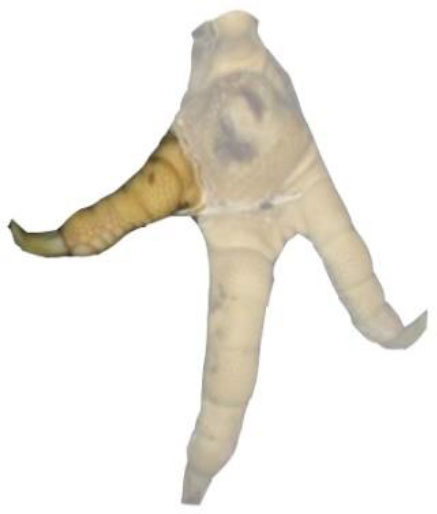	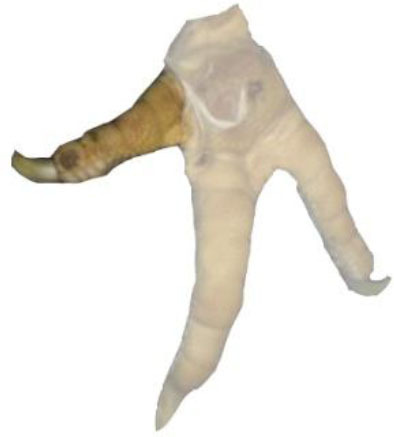	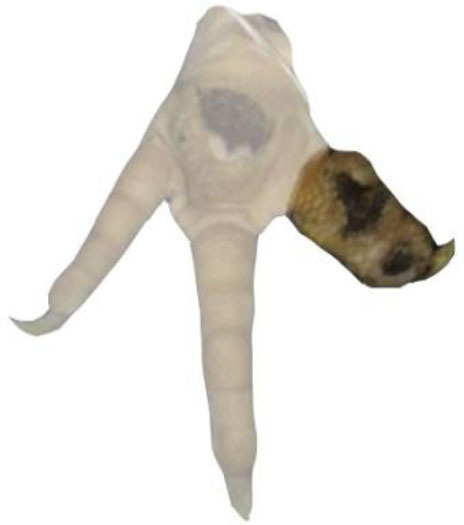	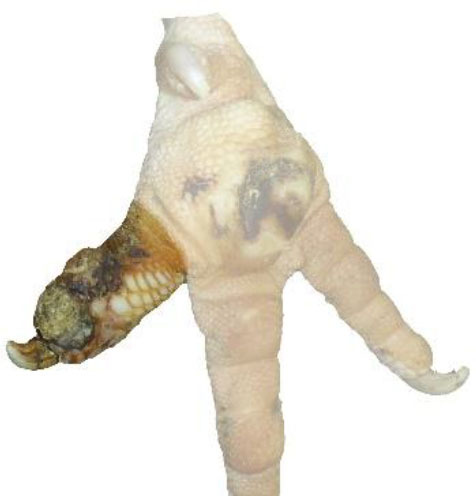
Intact digit	Small, punctual alterations, on 1–2 segments and/or hematomas	Small, punctual alterations on >2 phalanxes or altered lesion in one phalanx which covers <50%	Altered lesion in one phalanx which covers more than 50% of the phalanx or several altered lesions in >one phalanx which cover <50%	Altered lesion in > one phalanx which covers ≥ than 50% of each phalanx or altered lesions covering an area larger than the phalanx

*Digits (2–4) were scored separately, the digit used as example for the scoring system is highlighted (pictures taken by Jenny Stracke)*.

**Table 3:** Scoring system for the digital pads: Grade of swelling on the digital pad.

**Score 0**	**Score 1**	**Score 2**
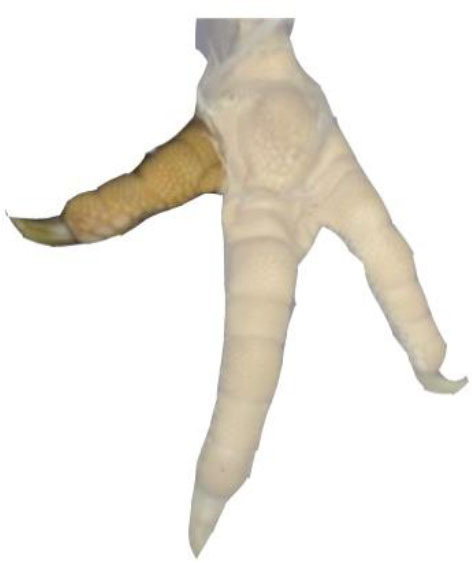	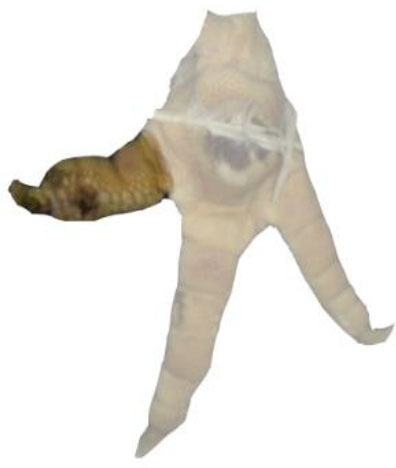	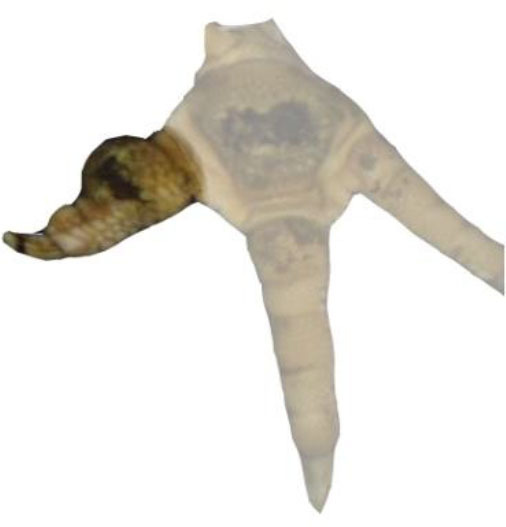
Intact digit	Slight swelling	Distinct swelling

*Digits (2–4) were scored separately, affected digit is highlighted (pictures taken by Jenny Stracke)*.

### Surface Measurements

A detailed survey of different parameters was conducted for 250 feet taken from the dataset. Here, the ImageJ software (Rasband, W.S., ImageJ, U. S. National Institutes of Health, Bethesda,MD, USA, https://imagej.nih.gov/ij/, 1997–2018) was used. Measurements were conducted to calculate the respective proportion of altered areas compared to different reference areas (RA). Therefore, the total foot (RA1), the metatarsal pad (RA2), the alteration on the footpad and the alterations on the digital pads (digital pad 2–4 in total) were tagged using the “freehand tool” in the software program (ImageJ, U. S. National Institutes of Health, Bethesda, MD, USA). RA1 included digital pads 2–4; the first digit was excluded as the suspension in the slaughter hooks did not allow a consistent presentation in the pictures of the automatic camera system. Furthermore, RA1 included the metatarsal pad, the remaining leg being excluded in the measurements (Figure 1). RA2 (metatarsal pad) was characterized by its kurtosis, the borders being specified at the start of the curvature (Figure 1). Alterations were defined as brownish discolorations, which could range from light to dark. The number of pixels on the surface area of both the reference and alteration was calculated to determine the respective proportion of the altered area compared to the respective reference (relative size of the lesion). All measurements were performed by one observer, observer reliability being ensured beforehand (see below for details). The percentage of the altered area in relation to the respective reference was calculated based on the following parameters:

- Alteration on the metatarsal pad in relation to the size of the metatarsal pad (standard)
- Alteration on the metatarsal pad in relation to the total foot (FP/RA1)
- Alteration on the digital pads in relation to the total foot (DP/RA1)
- Alteration on digital pads in relation to the metatarsal pad (DP/RA2)
- Alterations on metatarsal pad and digital pads in relation to the total foot (FULL)
- Size of the metatarsal pad in relation to the total foot (relative size of the metatarsal pad).

### Observer Reliability

A separate dataset of digital pictures was used to test the observer reliability of the scoring system for detecting FPD (400 feet from two flocks, male animals, left and right feet). This sample was evaluated by two experienced observers (researcher/veterinarian). The applied scoring system can be found in Table 1.

Observer reliability for scoring the digital pads was calculated using a random sample (100 pictures) taken from the original subset. This sample was evaluated by two experienced observers (researcher/veterinarian). The scoring system can be found in Tables 2, 3.

Observer reliability for the surface measurements was calculated using another dataset of 100 random pictures taken from the original dataset. Both observers (researcher) were experienced in using the program.

### Statistical Analysis

For the statistical analysis, the SAS software (V.9.4, Statistical Analysis Institute, Cary, NC, USA) was used.

Observer reliability was calculated using the Krippendorff's alpha “macro” developed by Hayes and Krippendorff (27). The Krippendorff's alpha is a reliability coefficient, which in contrast to other reliability coefficients (e.g., the prevalence-adjusted and bias-adjusted kappa) does not only includes perfect agreements, but also takes into account the degree of discrepancies. This means that, if the given score levels differ only slightly (e.g., by one scoring level), the result would turn out better than if the score level difference is more pronounced (e.g., more than one scoring level) (28). The respective data type (ordinal data for scoring data; metric data for the measurements using ImageJ) was taken into consideration. Each data set was calculated separately. Observer reliability was evaluated using the classification proposed by Landis and Koch (29) (<0.00 = poor; 0.00–0.20 = slight; 0.21–0.40 = fair; 0.41–0.60 = moderate; 0.61–0.8 = substantial; 0.81–1.00 = almost perfect).

Scoring of the digital pads was analyzed by descriptive analysis using the FREQ procedure in SAS. Furthermore, principal component analysis (PCA) was conducted to condense the different parameters to one “digital score” in order to allow a comparison with the standard FPD scoring system. The PCA is a tool in multivariate statistics used for exploratory data analysis. It is commonly used for dimensionality reduction, by projecting data points to a few principal components and therefore, for obtaining lower-dimensional data while preserving as much of the data's variation as possible. The PCA is normally based on a matrix of Pearson‘s correlation of the original data, assuming that the variables are continuous. As our model included ordinal variables as well, a polychoric matrix was calculated first to serve as basis for the PCA. Here, the CORR procedure was applied using the polychoric option. The PCA was performed with the FACTOR procedure with the following parameter settings: method=PRINCIPAL, priors=SMC, rotation=VARIMAX. The number of extracted factors was specified using the mineigen statement (minimum eigenvalue) which was set to 1, therefore retaining components with an eigenvalue of 1 or >1. Corresponding PC scores for each foot were finally calculated with the SCORE procedure.

To analyze the relation between scoring of the digital pads with the standard FPD scoring system, a correlation analysis was performed using the CORR procedure, calculating the Spearman correlation coefficient between the standard FPD scoring system and the best factor resulting from the PCA. Furthermore, a generalized linear mixed model (GLIMMIX procedure) was calculated for this factor, including flock as a random effect. The standard FPD scoring system and the interaction between flock and the standard FPD scoring system were included as fixed effects, pairwise comparisons being conducted using Tukey-Kramer tests.

To analyze differences between different digits regarding altered lesions and swellings, the Friedman test was calculated for each measurement separately using the ANOVA procedure (class variable: digital pads 2–4) in conjunction with the RANK procedure done by the blocking variable (foot).

The surface measurements were analyzed on a descriptive basis using the MEANS procedure in SAS. Furthermore, correlations were calculated using the CORR procedure, calculating the Pearson correlation coefficient between all parameters described above.

A generalized linear mixed model (GLIMMIX procedure) was calculated for the above mentioned parameters to analyze differences between the scoring levels of the standard scoring system, including the flock as random effect. Pairwise comparisons were conducted using Tukey-Kramer tests.

## Results

Observer reliability resulted in “moderate”—“almost perfect” results for all observations. Values of the Krippendorff‘s alpha coefficients for the different measurements can be found in Table 4.

**Table 4:** Observer reliability.

**Parameter**	**Krippendorff‘s alpha**
**Standard scoring system (*****n*** **= 400)**	
Digital pictures of standard FPD scoring system	0.70
**Scoring of the digital pads (*****n*** **= 100)**	
Altered lesions digital pad 2	0.82
Altered lesions digital pad 3	0.85
Altered lesions digital pad 4	0.84
Grade of swelling digital pad 2	0.61
Grade of swelling digital pad 3	0.80
Grade of swelling digital pad 4	0.67
Number of affected digits	0.83
**Surface measurement**	
Number of pixels on the total foot	0.97
Number of pixels on the metatarsal pad	0.84
Number of pixels of alterations on the metatarsal pad	0.83
Number of pixels of alterations on the digital pads (total)	0.69

### Scoring FPD of the Digital Pads

**Figure 2:**
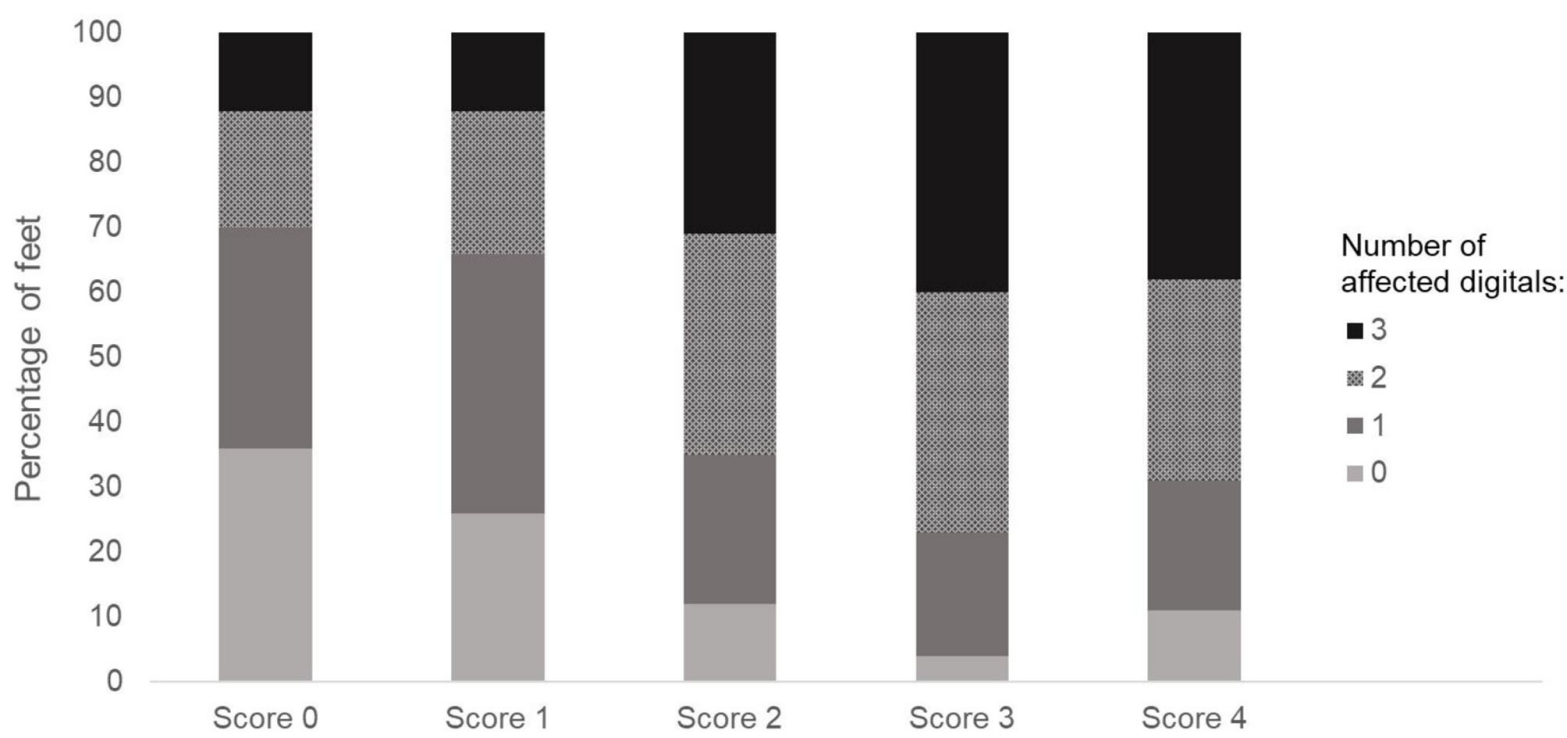
Number of affected digits for the different scoring levels of the standard FPD scoring system of the metatarsal pad (score 0 = intact foot; score 1 = alterations ≤10%; score 2 = alterations ≤25%; score 3 = alterations ≤50% and score 4 = alterations >50%). Data are presented as percentage, *n* = 500 feet.

In total, 17.8% of all feet (*n* = 500) were found to have intact digitals. In 27.2% of the feet, alterations and/or swelling became obvious on one digit, 28.4% of the feet showed alterations and/or swelling on two digits and in 26.6% of the feet, three digits were affected. Figure 2 presents the results found for the number of affected digital pads per scoring level of the standard scoring scheme. Feet with the standard scoring level 0 (*n* = 100) were found to have one affected digital pad in 34.0% of the cases, 18.0% were found to have incidents on two digits, and in 12.0% of the cases, all three digits were affected. Scoring level 1 of the standard FPD scoring system was found to include 26.0% intact digits, in 40.0% of the cases, one digit was affected, whereas 22.0% of the cases showed alterations on two digits and in 12.0 % of the cases, alterations on three digital pads. In scoring level 2, the percentage of intact digital pads decreased to 12.0%, 23.0% of the feet were found to show alterations on one, 34.0% thereof on two and 31.0% on three digits. Scoring level 3 revealed 4.0% of the feet to have intact digital pads, with 19.0% of the feet showing alterations on one, 37.0% thereof alterations on two and 40.0% alterations on three digital pads. Results for scoring level 4 were similar, with 11.0% of the feet having no affected digit, 20.0% thereof one, 31.0% two and 38.0% three affected digital pads.

**Figure 3:**
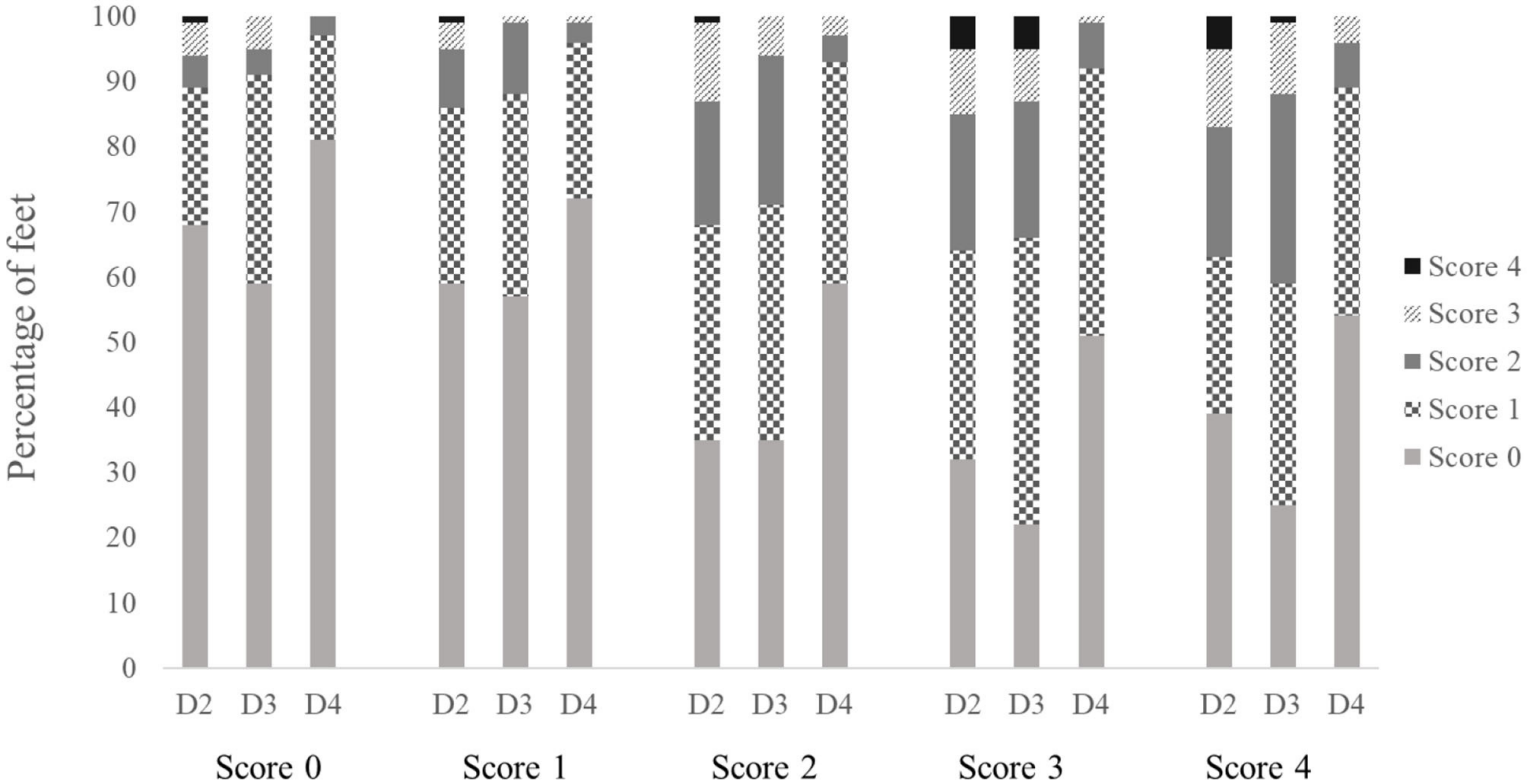
Altered lesions on digital pads for the different scoring levels of the standard FPD scoring system of the metatarsal pad (score 0 = intact foot; score 1 = alterations ≤10%; score 2 = alterations ≤25%; score 3 = alterations ≤50% and score 4 = alterations >50%). Digits were scored separately (D2–D4). Data are presented as percentage, *n* = 500 feet.

Figure 3 shows the results for the altered lesions found on each digit (D2–D4) in the different scoring levels of the standard FPD scoring system of the metatarsal pad. If affected, most lesions were allocated the severity grade 1 of the digital pad scoring system (standard FPD score: 0: 16–32%; 1: 24–31%; 2: 33–36%; 3: 32–44%; 4: 24–35%). Generally, severity grades of the digital score increased with an increasing severity in the standard FPD scoring system. However, differences between the standard FPD scoring levels 0 and 1 and between levels 2, 3 and 4 were only marginal (digital scoring levels for the standard score 0: 3–11%; 1: 4–14%; 2: 7–32%; 3: 8–36%; 4: 11–41%).

The results of the Friedman test revealed a significant difference between the digits [*F*_(2,1,461)_ = 65.55; *p* < 0.001], with digit 4 being affected least.

This could also be found for the grade of swelling [*F*_(2,1,461)_ = 184.89; *p* < 0.001]; here, digit 2 was affected most. Regarding the process of scoring, results revealed differences in the observer reliability, with the best values found for digit 3, whereas scoring for digit 2 and 4 only achieved moderate results.

**Figure 4:**
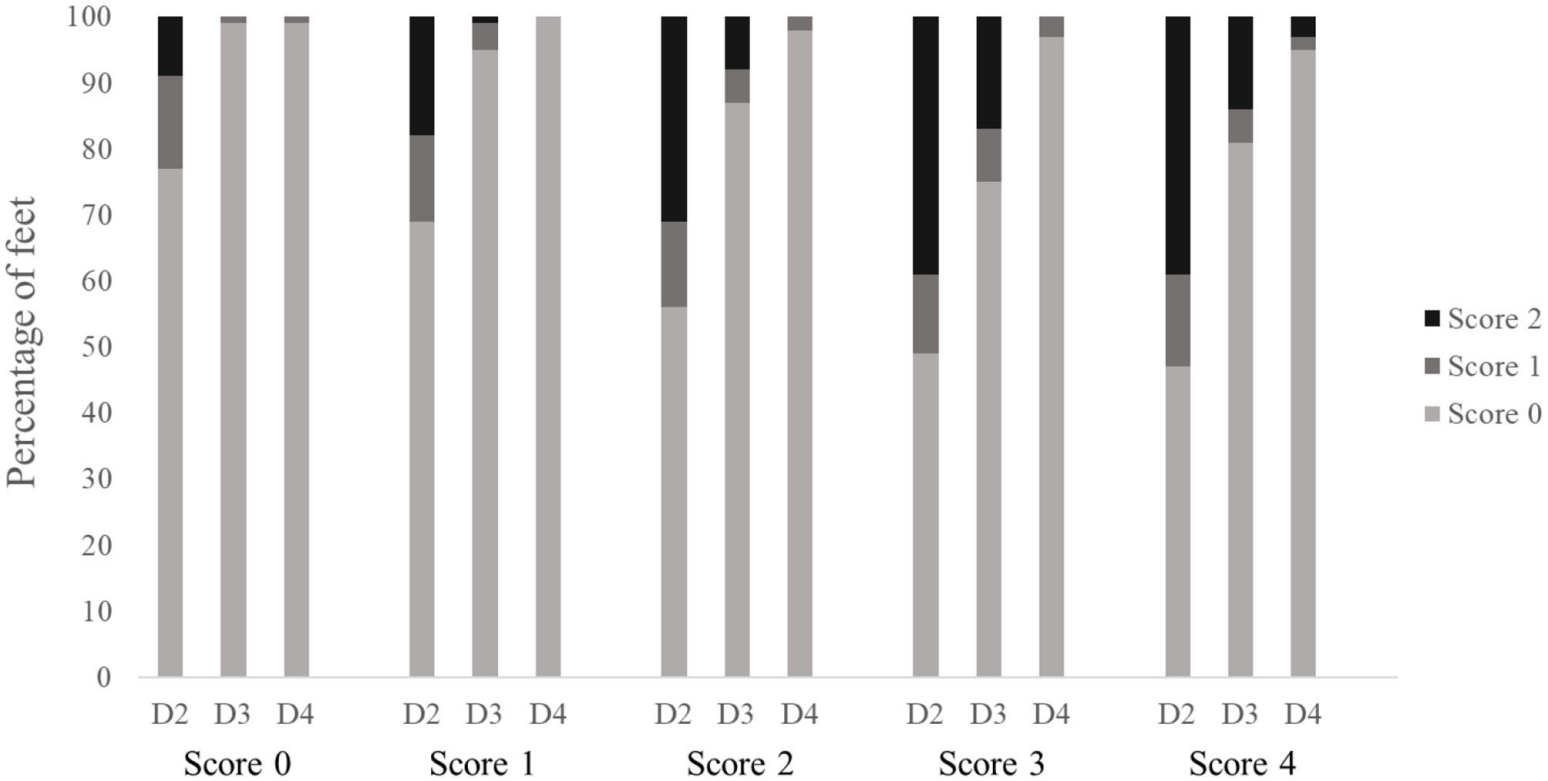
Grade of swelling on digital pads for the different scoring levels of the standard FPD scoring system of the metatarsal pad (score 0 = intact foot; score 1 = alterations ≤10%; score 2 = alterations ≤25%; score 3 = alterations ≤50% and score 4 = alterations >50%). Digits were scored separately (D2–D4). Data are presented as percentage, *n* = 500 feet.

Figure 4 presents the results for the grade of swelling found for each digital pad. Here again, severity grades of the digital score increased with an increasing severity in the standard FPD scoring system.

**Figure 5:**
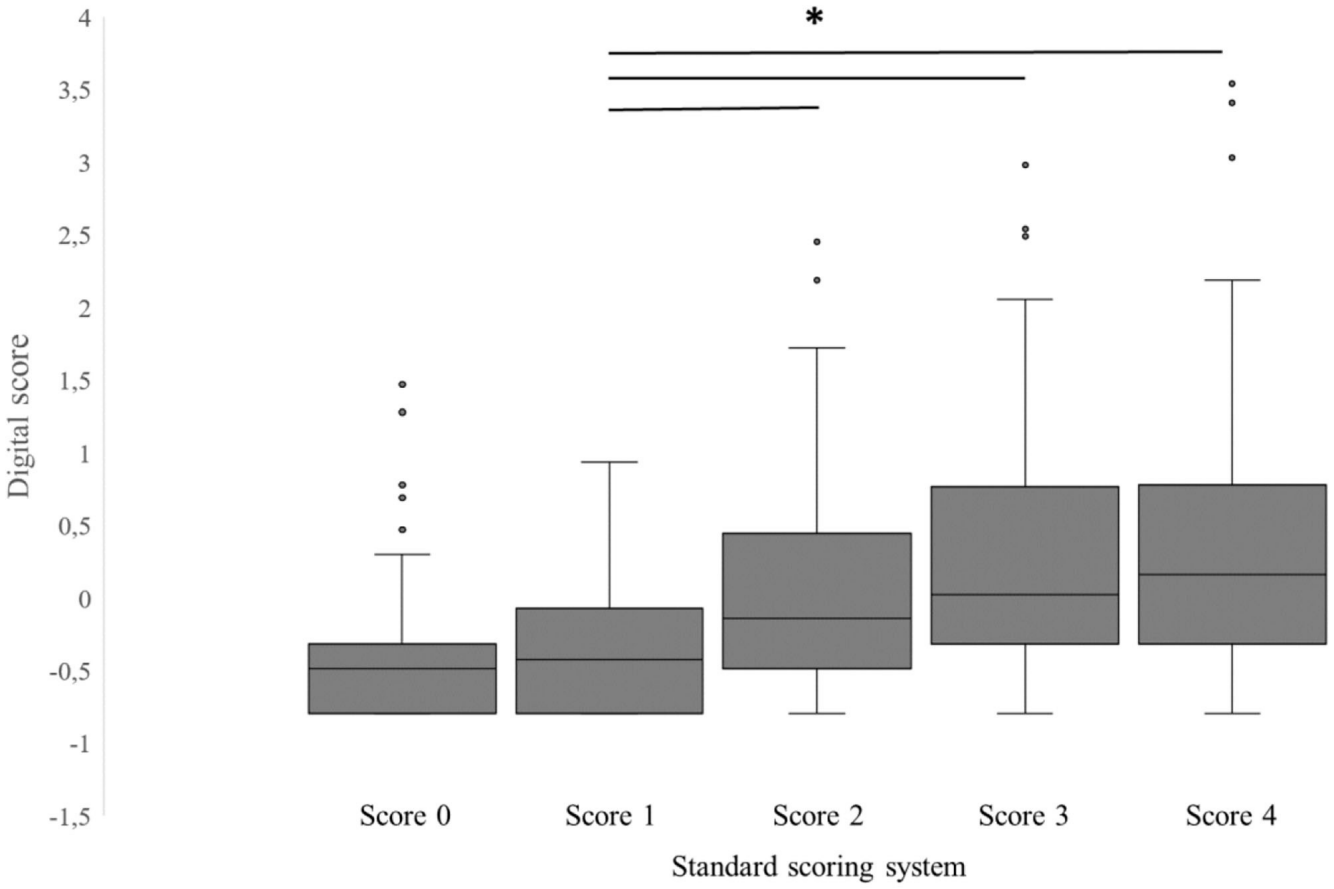
Boxplots of the digital score [factor revealed from principal component analysis (PCA)] vs. the standard FPD scoring system of the metatarsal pad (score 0 = intact foot; score 1 = alterations ≤10%; score 2 = alterations ≤25%; score 3 = alterations ≤50% and score 4 = alterations >50%). Data are presented as boxplots (data range, median, lower quartile, and upper quartile; outliers are included in the graph as dots), *indicates a *p* < 0.05, *n* = 500 feet.

The PCA resulted in one factor with an eigenvalue >1 (2.8). Using this factor per foot (hereafter denoted as digital score) and the respective scoring level of the standard FPD scoring system for the correlation analysis revealed a moderate and significant positive correlation (r_s_ = 0.41; *p* < 0.001). A significant difference between scoring levels of the standard FPD scoring system could be found for the digital score [*F*_(4,448)_ = 6.4; *p* < 0.001], whereas pairwise comparisons revealed higher levels in scoring levels 2–4 compared to scoring level 1, whereas no significant differences could be found for the remaining combinations (Figure 5). Furthermore, a significant effect could be found for the interaction of the standard FPD scoring system and the flock [*F*_(35,448)_ = 5.3; *p* < 0.001]. Pairwise comparisons resulted in differences between particular flocks for the standard FPD scoring levels 2–4 (all *t* < 8.0; all *p* < 0.05), whereas no differences were found for scoring levels 0 and 1.

### Surface Measurements

**Figure 6:**
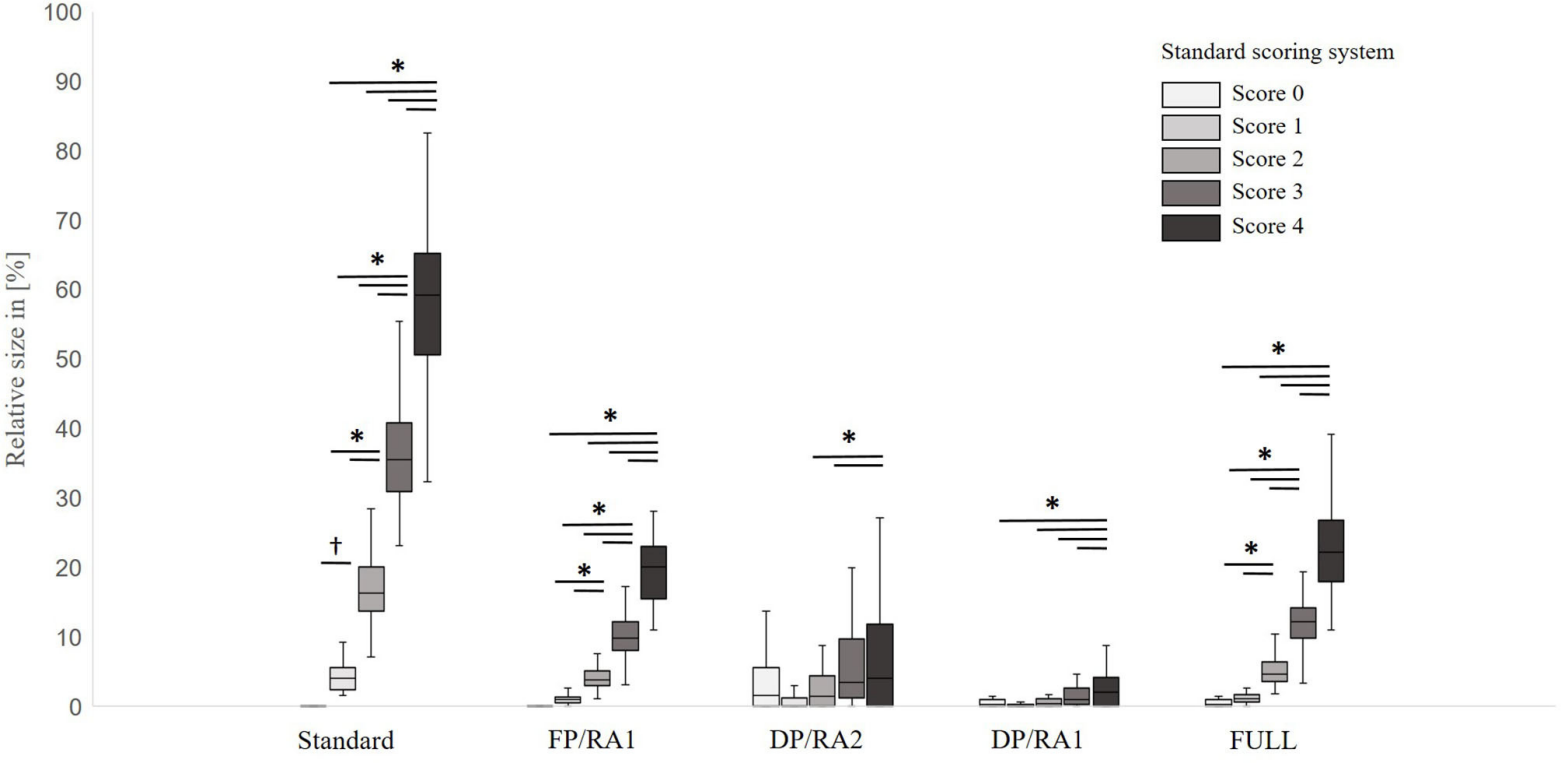
Boxplots of the relative size of alterations measured for the different parameters by scoring level of the standard FPD scoring system. Data are presented as boxplots (data range, median, lower quartile and upper quartile; outliers are not included in the graph); *n* = 250, (Standard, alteration on the metatarsal pad in relation to the size of the metatarsal pad; FP/RA1, alteration on the metatarsal pad in relation to the total foot; DP/RA2, alteration on digital pads in relation to the metatarsal pad; DP/RA1, alteration on the digital pads in relation to the total foot; FULL, alterations on metatarsal pad and digital pads in relation to the total foot), *indicates a *p* < 0.05,^†^indicates a *p* < 0.1.

The results of the surface measurements can be found in Figure 6. For all parameters, a significant difference between scoring levels of the standard FPD scoring system could be found [standard system: *F*_(4,221)_ = 493.8; *p* < 0.001, FP/RA1: *F*_(4,229)_ = 190.7; *p* < 0.001), DP/RA2: *F*_(4,222)_ = 4.3; *p* < 0.01, DP/RA1: *F*_(4,229)_ = 6.6; *p* < 0.001, FULL: *F*_(4,229)_ = 211.9; *p* < 0.001]. Pairwise comparisons revealed significant differences between all scoring levels for the standard FPD scoring system (all *p* < 0.05), except for the scoring level 0 vs. scoring level 1. Here, a tendency could be found (*p* = 0.056). In FP/RA1, no significant differences between scoring levels 0 and 1 (*p* = 0.53) in contrast to all other combinations (all *p* < 0.05) were found. In DP/RA2, significant differences were found for the pairing of scoring levels 4 and 2 and scoring levels 4 and 1 only (all *p* < 0.5). For DP/RA1, significant differences were restricted to the differentiation between scoring level 4 and respective scoring levels (all *p* < 0.05). The FULL scoring system found no significant differences between scoring levels 0 and 1 (*p* = 0.99) in contrast to all other combinations (all *p* < 0.05).

The correlation coefficients between all parameters and the *p*-values of the correlation can be found in Table 5. A strong positive correlation was found between the standard FPD scoring system and alterations measured on the metatarsal pad in relation to RA1 (*p* < 0.001; r_p_0.9) and between the standard system and the FULL scoring system (*p* < 0.001; r_p_ = 0.9). The positive correlation between the standard FPD scoring system and measurements on the digital pads were only weak (r_p_ = 0.2 and 0.3; both *p* < 0.001).

**Table 5:** Correlation analysis between parameters of the surface measurement.

**Parameters**	**r_**p**_**	***p*-value**
**Standard with**		
FP/RA1	0.93	<0.001
DP/RA1	0.31	<0.001
DP/RA2	0.23	<0.001
FULL	0.91	<0.001
**FP/RA1 with**		
DP/RA1	0.17	<0.01
DP/RA2	0.09	*P*=0.17
FULL	0.92	<0.001
**DP/RA1 with**		
DP/RA2	0.84	<0.001
FULL	0.53	<0.001

*The Pearson correlation coefficient (r_p_) is presented. Standard, alteration on the metatarsal pad in relation to the size of the metatarsal pad; FP/RA1, alteration on the metatarsal pad in relation to the total foot; DP/RA1, alteration on the digital pads in relation to the total foot; DP/RA2, alteration on digital pads in relation to the metatarsal pad; FULL, alterations on metatarsal pad and digital pads in relation to the total foot*.

The scoring of the digital pad in relation to the FULL scoring system resulted in a moderate and significant positive correlation (*p* < 0.001; r_p_ = 0.5).

**Figure 7:**
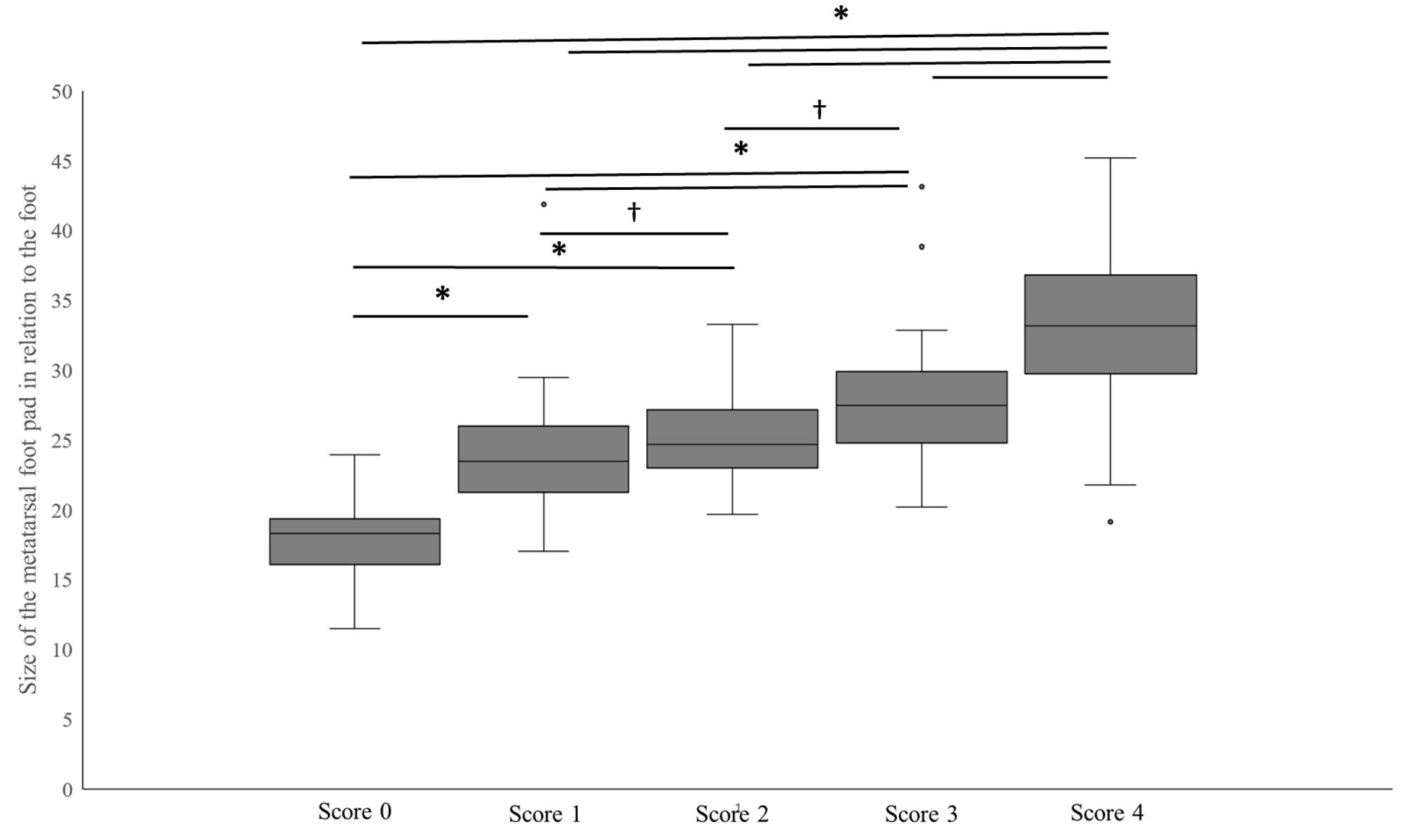
Size of the metatarsal pad (independent of alterations) in relation to the size of the total foot for the scoring levels of the standard FPD scoring system. Data are presented as boxplots (data range, median, lower quartile, and upper quartile), *indicates a *p* < 0.05,^†^indicates a *p* < 0.1, *n* = 242 feet.

The results for the relative size of the metatarsal pad revealed a significant difference between the scoring level of the standard FPD scoring system [*F*_(4,222)_ = 59.6; *p* < 0.001] (see Figure 7). The size increased significantly between severity grades (all *p* < 0.05), except for the comparison of scoring levels 1 and 2 (*p* = 0.09) and 2 and 3 (*p* = 0.08) where a tendency could be found.

## Discussion

The aim of this study was to evaluate alterations on the digital pads of turkeys and to compare the severity to those of alterations assessed on the metatarsal pads, evaluated by a standardized scoring system for FPD (11).

Even if included in some of the studies evaluating FPD in turkeys (1) and in assessment schemes in Germany (6), the digital pads are currently not included in the evaluation when using standard scoring systems for practical applications (9, 11). In contrast to turkeys, scoring systems used in broilers partially include lesions on the digital pads (20) though only in the highest severity grades. According to the literature (11, 21, 22), the assumption was that with rising severity grades of altered lesions on the metatarsal pad, alterations on digital pads would increase alike. If this would have been the case, the metatarsal pad could be used as a representative for the whole foot.

In contrast to the current opinion, the results of this study could not confirm a homogeneous representation of the severity of FPD when considering metatarsal pads and digitals separately. Even if the study generally found higher severity scores for lesions, swellings, the number of affected digits and the combination of all those parameters for the higher scoring levels of the standard FPD scoring system, no clear distinction became obvious between scoring levels 0 and 1 or between scoring levels 2–4. These findings were also confirmed by the surface measurements. When measuring the alterations on the digital pads, no differences in size could be found between standard scoring levels 0 and 1; numerical data even found higher values for scoring level 0 compared to scoring level 1. Furthermore, no differences in the size of the alteration were found between standard scoring levels 2 and 3. Additionally, correlations between measurements based on the standard system and measurements on the digital pads were only weak—also indicating a rather asynchronous occurrence of alterations on digital pads and the metatarsal pad. Especially alterations on digital pads of feet, which were scored as intact, according to the standard FPD scoring system, is relevant with regards to animal welfare. This effect could be due to missing parameters in the standard system. Stracke et al. (14) were able to demonstrate that other parameters like perivascular pododermatitis and re-epithelialized granulation tissue occur in feet scored as potentially intact, too. The occurrence of alterations on the digital pads therefore could be an effect of previous (old) metatarsal pad injuries. However, we are unable to ascertain which part of the foot was affected first. Further studies are needed to clarify the roots of this problem.

The most important reason for FPD in turkeys is the litter quality, with wet litter facilitating its occurrence (1–4). Feet evaluated in this study were picked randomly from a subset of 16 flocks; information on husbandry and litter quality of these flocks was not evaluated. Furthermore, standard FPD scores could not be streamlined for the different flocks, as not all scoring levels were present in all flocks. Therefore, it cannot be excluded that some effects found in the present study were due to varying husbandry conditions or restricted to specific flocks. The interaction effect between the flock and the standard FPD scoring system on the digital score, which could be found in this study, indicates no differences between flocks for standard scoring levels 0 and 1, whereas in standard scoring levels 2–4, differences between single flocks were present. However, more information on the background of flocks would be necessary to give a comprehensive picture in this case and to examine the causes of alterations on the digitals. This study only presents one first analysis of alterations on digitals and their linkage to a standard scoring system measuring the alterations on the metatarsal pad. Independent of the origin, the heterogeneous occurrence of alterations on digitals and metatarsal pads should be one hint, that scoring the metatarsal pads solely, might be insufficient to assess animal welfare.

Apart from that, while scoring the digital pads in this study, there was the subjective impression of finding a higher incidence of dirt under the nails of the animals showing higher severity grades in FPD scoring on the digital pads. However, we did not systematically evaluate this effect, and were unable to provide any information on correlations between dirtiness of the feet and FPD on the digital pads.

Both, metatarsal pad and digital pads are equipped with special fat structures (*Corpora adiposa plantaria superficialia et profunda*) (30, 31), which serve as mechanical protection from external pressure. These fat structures are more pronounced on the metatarsal pad; consequently, on the digital pads, there is less protection to the bones and underlying structures. It might therefore be plausible that birds start to relieve the digital pads by putting weight on the metatarsal pads instead when the digits are affected. This could be one explanation for the inconsistent development of alterations between the digital and metatarsal pads. However, this is highly speculative as there are no existing studies, neither evaluating the gait patterns of turkeys due to FPD in detail, nor the pressure load on specific body parts. There are results in laying hens providing evidence that there is a genetic influence on pressure load, which might be due to different weights of the animals (32), which might be evident in turkeys also (33, 34). That the pressure load might play a role in the occurrence of FPD on the digits is substantiated by the differences found between the digital pads concerning the alterations (digit 4 affected the least) and the grade of swelling (digit 4 affected the least, digit 2 affected the most). Both indicate an imbalanced pressure regarding the total foot. One explanation for this effect might be the leg position of the animals. Turkeys tend to be slightly bow-legged, leg problems like varus or valgus deviations can occur with a high prevalence, as a Danish study showed (35). Varus deviations were found to be correlated to weight in broiler chickens (36, 37), similar results being found for turkeys, too (38). We did not evaluate the weight of the animals in our study, but further studies might be beneficial in providing evidence on the development of FPD, including different gait patterns (e.g., by measuring the pressure load on specific body parts) and influences on other health parameters like for example weight gain (33, 34).

Generally, FPD is assumed to be painful (17, 39–41). However, relating the results of these previous studies to FPD by mainly using the gait score as parameter is not easy—other pathologies inducing an impaired gait, such as femoral head necrosis (42, 43) or osteomyelitis (44), being common in fast-growing poultry, too. Hocking et al. (45) could not prove higher FPD scores to be linked to painfulness in turkeys in a pharmacological study. In the context of pain, the grade of swelling might be a valuable parameter, as swelling is associated with inflammatory processes. Inflammation then again can be assumed to be linked to pain (46). Nevertheless, evaluating swelling from digital pictures as done in the presented study has to be interpreted with caution. As the results from the observer reliability confirm, the evaluation of the occurrence of swelling and the differentiation between slight and distinct was subjective. Defining the normal size and specified deviations from the norm proved to be extremely difficult due to differing size of feet and digits. In this case, other methods might be more feasible, like the manual palpation of fluctuation or thermal measurements, even if those techniques cannot be applied at the slaughterhouse. Apart from the grade of swelling, the present study found lesions in 82.2% of the analyzed feet. The study by Stracke et al. (14) was able to show that small lesions on the metatarsal pad can already be characterized by ulcerations; this is most likely to be the case for the lesions found on the digital pads as well. In mammals, ulcerations are referred to as being painful [see (47) for a review in pigs]. Therefore, the occurrence of ulcerations (on both, metatarsal pad and digital pads) should be considered a welfare aspect in turkeys, too [i.e., the concept of the Five Freedoms (Freedom from pain, injury and disease (48))].

In order to improve the assessment scheme for FPD with regard to animal welfare, it might be beneficial to include the monitoring of alterations on the digital pads. The present study found a high correlation between the FULL scoring system (including the digital pads) and the standard FPD scoring system; using the FULL system might therefore be an adequate alternative to the sole usage of the metatarsal pad. One critical point would be the easy application of the scoring system in situations where scoring has to be conducted fast, as scoring the total foot seems to be more demanding than simply scoring percentages on the metatarsal pad. However, observer reliability of alterations on the digital pads in the present study were good up to nearly perfect, implicating a good reliability of the scoring system *per se*. As an alternative, evaluating FPD on the digital pads could be conducted as part of an extended standard FPD scoring system. The involvement of digital pads could be implemented as an additional binomial score (alterations: yes/no) for all severity classes. This could also be beneficial with regard to the upcoming automatic assessment of FPD using 2-D-RGB-image analysis at the slaughterhouses. Even if the inclusion of scoring on the digits should be easy to apply, a separate evaluation of the digital pads would ease the manual evaluation in case of a technical breakdown. Besides implementing the digits, using the total foot as reference could refine existing automatic assessment methods, especially regarding recent discussions on the correct definition of the size of the metatarsal pad (13). As the results in this study provide strong correlations between the standard FPD scoring system and FP/RA1 (using alterations on the metatarsal pad in relation to the total foot), this could be a good alternative. Furthermore, the present study found the size of the metatarsal pad to increase with an increase in severity of the standard scoring. Similar effects were found in a study by Klambeck et al. (49) examining FPD in ducks. This effect might be due to inflammatory processes. Either way, whatever the underlying causes might be, such effects can falsify the assessment of FPD, when the reference for the assessment is based on the size of the metatarsal pad only. Using the total foot as a reference would not prevent such negative effects of swelling occurring, but could minimize the error rate. Furthermore, keeping the rapid development of automatic systems in mind, it might be worth thinking of possibilities of integrating the grade of swelling as well to gain a comprehensive picture of the pathology.

## Conclusion

To conclude, the present study found no equivalent occurrence of alterations on the digital pads compared to alterations on the metatarsal pad assessed by a standard five-point scoring system. Pathologic alterations on the digital pads were present at standard scoring levels 0 already; no differentiation became obvious between the higher standard scoring levels 2–4. Good correlations were found when comparing the standard FPD scoring system to a system including alterations on the digital pads. Therefore, the authors state that including the digits could improve the present system with regard to animal welfare.

## Data Availability Statement

The raw data supporting the conclusions of this article will be made available by the authors, without undue reservation.

## Ethics Statement

Ethical review and approval was not required for the animal study because no living animals were included in this study. This study presents results based on digital pictures of turkey feet, samples base on recordings which were conducted during the standard process at the slaughterhouse.

## Author Contributions

BS and NK were responsible for financial acquisition and project development. JS, BS, and NK contributed to the conception and design of the present study. BS and JS were the principal investigators for this project. JS, NV, FM, and SD participated in data collection and performed the data analysis. JS designed and performed the statistical analysis. All authors were involved in interpreting the results and in drafting the manuscript.

## Conflict of Interest

The authors declare that the research was conducted in the absence of any commercial or financial relationships that could be construed as a potential conflict of interest.
